# Phospholipid-independent biogenesis and function of the RP4 conjugation pilus

**DOI:** 10.1038/s41467-026-74409-x

**Published:** 2026-06-17

**Authors:** Naito Ishimoto, Shan He, Mikhail Bogdanov, Terry K. Smith, Gad Frankel, Konstantinos Beis

**Affiliations:** 1 Department of Life Sciences, Imperial College London, London, UK; 2 Rutherford Appleton Laboratory, Research Complex at Harwell, Didcot, Oxfordshire, UK; 3 Department of Biochemistry & Molecular Biology, University of Texas-Houston, McGovern Medical School, Fannin, USA; 4 BSRC, Schools of Biology and Chemistry, University of St Andrews, St Andrews, UK; 5 Present Address: Drug Design Laboratory, Graduate School of Medical Life Science, Yokohama City University, Tsurumi, Yokohama, Japan

**Keywords:** Cryoelectron microscopy, Bacteriology, Bacterial structural biology

## Abstract

Bacterial conjugation, the process of horizontal gene transfer between bacteria, is initiated by mating pair formation (MPF) via a conjugative pilus. Conjugation of the IncP RP4 plasmid is mediated by short mating pili. Here, we report the cryo-EM structure of the RP4 pilus at 2.74 Å resolution. Uniquely, both the structural and quantitative mass spectral analyses revealed that the cyclic TrbC pilin subunit is not lipidated. Consistently, an *E. coli pgsA* mutant lacking phosphatidylglycerol (PG) can serve as a donor of RP4 but not of F- (pKpQIL), H- (R27) or W- (R388) pili, whose biogenesis and DNA transfer is PG-dependent. RP4 is the first example of a lipid-independent functional mating pilus. This discovery suggests that an amphipathic lipid moiety is not universally essential for the biogenesis of conjugative pili and MPF, providing an alternative model for their assembly and function. These data expand our understanding of the diverse bacterial mechanisms employ to transfer genetic material.

## Abstract (web summary)

Bacteria can exchange plasmids via conjugation using filamentous appendages, known as pili, containing lipidated subunits. Here, the authors elucidate the structure of a conjugative pilus and show that, unlike other pili, it is built from non-lipidated cyclic subunits, suggesting pilus assembly and DNA transfer can occur independently of lipids.

## Introduction

Bacterial conjugation^1^^,^^2^, an efficient mechanism of horizontal gene transfer playing a central role in the dissemination of antibiotic resistance, relies on extracellular appendages known as conjugative pili (mating pili); these dynamic filaments mediate intercellular contacts between donor and recipient bacteria and facilitate DNA transfer^3^. Studies of the archetypical F-pilus, encoded by the pOX38 and pED208 plasmids, provided detailed molecular insights into the pilus biogenesis process^4–6^. Briefly, the TraA pilin, which is synthesized as a pre-pilin, is cleaved in the cytosol by the signal peptide peptidase LepB before it is inserted into the inner membrane (IM), where it accumulates as a reservoir. While extracted from the IM, the pilin co-extracts phosphatidylglycerol (PG). The pilin-PG complex then polymerizes into a helical filament on top of the type IV secretion system (T4SS), a multi-protein complex spanning the inner and outer membranes and mediating plasmid transfer^3–6^. Structural studies of the pOX38/pED208/pKpQIL-(IncF)^4^^,^^7^, R388- (IncW)^8^, and pKM101- (IncN)^9^ encoded pili revealed that they are hollow helical tubes, approximately 8-10 nm in diameter, with a central lumen of 2.5–3 nm, where the relaxase-bound single-stranded (ss) DNA can transverse^10^. The pilins are composed of three α-helices, α1–α3, connected by short loops; the helices display conformational variations in different pili. All known conjugative bacterial pili, the archaeal import pili^11^ and the T-pilus encoded by the Ti-plasmid of *Agrobacterium tumefaciens*^9^^,^^11^^,^^12^ have a bound phospholipid (e.g., phosphatidylglycerol (PG), phosphatidylethanolamine (PE), phospholipid GDGT-0, phosphatidylcholine (PC), and C_25_-C_25_, C_25_, C_25_ diether lipids) at a 1:1 stoichiometry with the pilin subunit. In the retractable F-pilus, the phospholipid acyl chains are parallelly arranged^4^, while in the unretractable R388 pilus, they are splayed^8^. Cryo-electron microscopy studies have shown that each TraA pilin subunit in the pilus is associated with a single PG molecule^4^. The PG molecule makes extensive contact with five surrounding TraA subunits, while each TraA subunit interacts with five PG molecules. Disrupting the interface between the PG and the pED208 TraA pilin by site-directed mutagenesis aborted pilus biogenesis^4^. The phospholipids act as a “molecular glue,” stabilizing the protein-protein interfaces and strengthening the entire filament. This intricate network of both protein-protein and protein-lipid interactions is crucial for assembling the helical filament. Stoichiometrically arranged PG molecules line the TraA pilin’s lumen with their solvent-exposed head groups directed to the interior of the pilus and the acyl chains entirely buried between protein subunits. The negatively charged PG molecules lining the pilus lumen have been suggested to neutralize the positive charge of the pilus lumen, thus providing directionality for electronegative ssDNA transport along the pilus interior^4^. The exception is the T pilus, where the PE contributes to the positive charge within the pilus lumen^9,11^.

We have recently reported a deviation from the prevailing model, showing that the TrhA pilin, encoded by the prototype IncHI1 plasmid R27, undergoes posttranslational C-terminal cleavage and cyclization^13^. Pilin cyclization is mediated via an intramolecular covalent head-to-tail peptide bond between Gly1 and Asp69, forming a macrocyclic structure. The cyclic pilin is associated with a PG moiety, and the overall pilus cryo-EM structure resembles the archetypical F-pilus^4^. We have recently shown that while Gly1 and Asp69 are conserved in IncH plasmids, cyclization of TrhA is promiscuous. Moreover, Asp69 is exposed on the mating pilus surface, conferring a negative charge; Asp69Arg and Asp69Lys substitutions confer positive surface charge, abrogating conjugation, which could be rescued using a recipient lacking PE^14^. Like the R27 TrhA pilin, it was previously shown biochemically that the RP4 (IncP) plasmid-encoded TrbC pilin is also cyclic^15^^–^^17^.

Several studies provided detailed molecular insights into the RP4 pilus biogenesis process^15^^[–17^. Following removal of the signal peptide by LepB, maturation of TrbC requires a yet unknown chromosome-encoded protease, cleaving the C-terminal 27 residues, and the plasmid-encoded TraF, which, while cleaving four additional C-terminal residues, cyclizes TrbC by forming a peptide bond between Ser1 and Gly78 of the mature pilin^15^^[–17^. Here, we report the cryo-EM structure of the RP4 pilus, which unexpectedly revealed that the cyclic TrbC pilin assembles into a pilus in the absence of phospholipids. Although the cyclic structure of the pilin has been proposed to provide the pilus with greater stability, this discovery provides an alternative model of pilus formation, lipid-independent pilus biogenesis, that diverges from the canonical model, where an amphipathic lipid moiety was considered essential for the assembly and stability of mating pili.

## Results and discussion

### The cyclic TrbC pilin is assembled into a mating pilus in the absence of a phospholipid

**Fig. 1:**
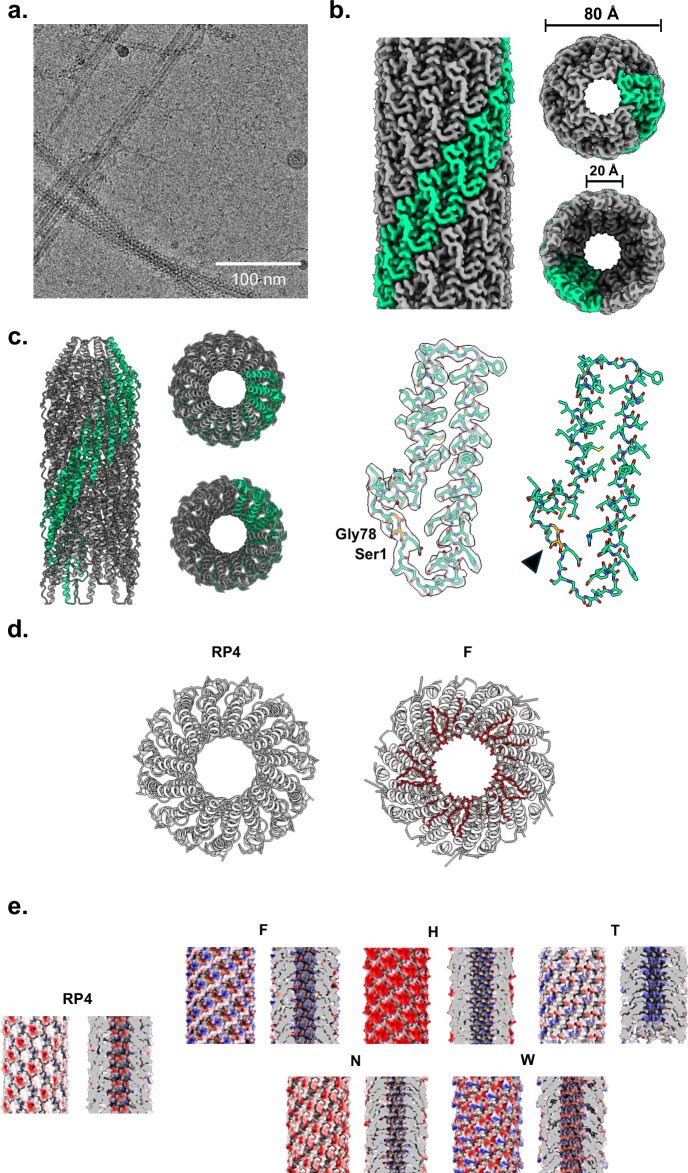
Cryo-EM structure of the RP4 pilus. **a** Representative micrograph of the straight RP4 pilus. Data are representative of *n* = 3 independent purifications. **b** Cryo-EM map of the RP4 pilus. One right-handed 5-start strand is shown in green and the rest of the filament in gray. The pilus has an outer diameter of ~80 Å and an inner lumen diameter of ~20 Å. **c** Atomic model of the RP4 pilus (left panel). The TrbC pilin is cyclic and lacks a bound phospholipid as revealed by the Coulomb map. The site of cyclization is indicated by an arrowhead and the residues in orange. **d** The F-pilus lumen is lined by phosphatidylglycerol (PG) (indicated by red sticks) compared to the RP4 pilus. **e** The RP4 pilus lumen displays a strong electronegative charge compared to the other bacterial pili that appear more neutral, whereas the T pilus has an alternating positive and negative charge. The exterior of the different pili displays variable charge profile.

**Fig. 2:**
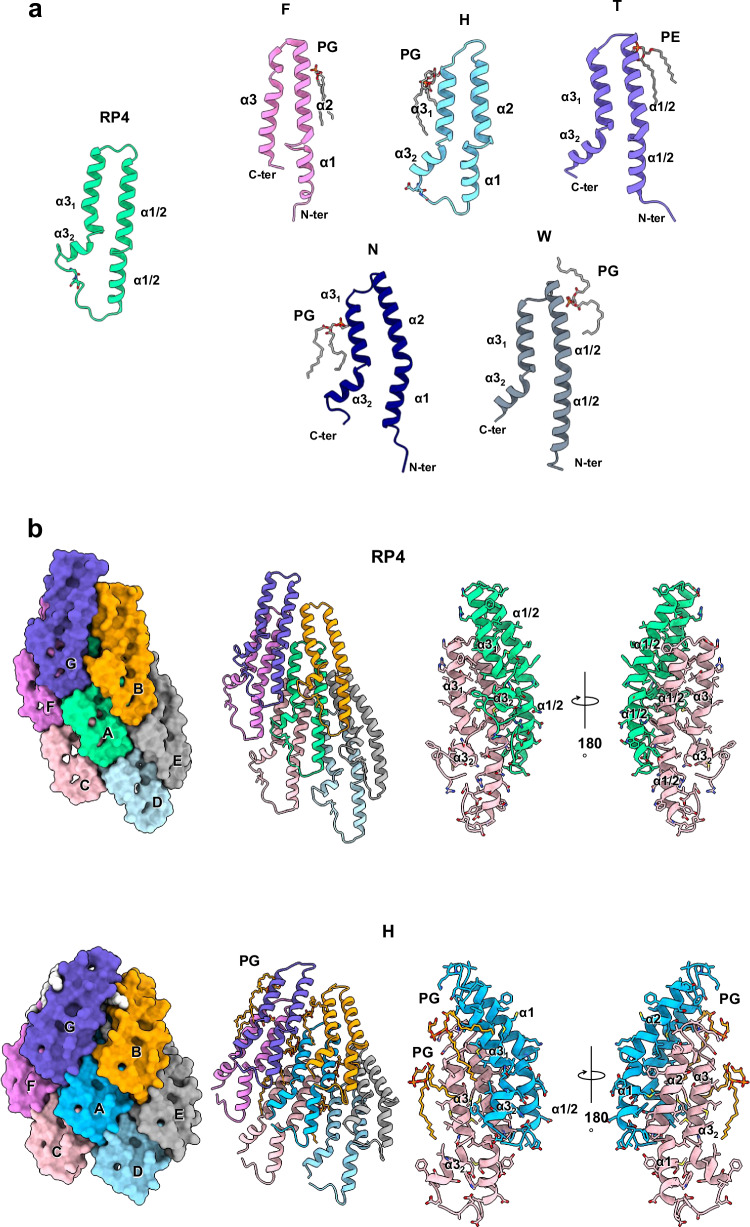
Comparison of the TrbC with other conjugative pilins. **a** The conformation of TrbC pilin resembles the VirB2. Pilins of different conjugative pili bind diverse phospholipids, phosphatidylethanolamine (PE) or phosphatidylglycerol (PG), and in different conformations. The pilin structures and their associated lipids are shown in cartoon and sticks, respectively. **b** In the absence of phospholipids, the neighboring TrbC protomers occupy the empty lipid-space to make extensive stabilizing interactions (top row). The lipidated TrhA is further stabilized by interactions with the PG lipid (bottom row). Each pilin protomer is shown in either surface or cartoon representation and colored differently.

**Fig. 3:**
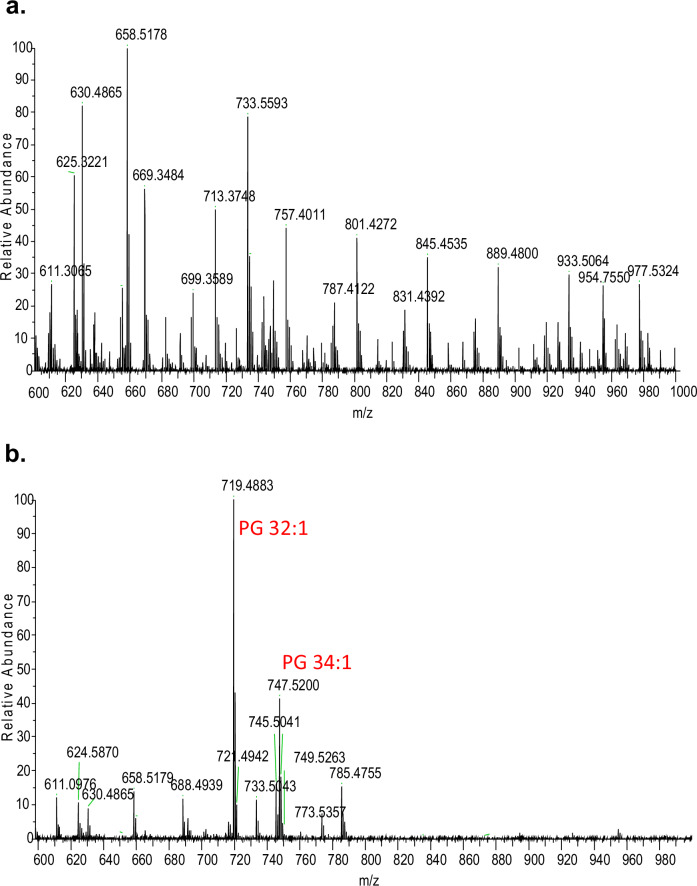
Mass spectrometric analysis of lipids associated with purified pili. Negative ion mode survey scans (600–1000 m/z) of lipids isolated from pili after treatment with phospholipase A2 (Supplementary Fig. 3 shows the 100–1000 m/z). Annotated phospholipid identities (phosphatidylglycerol (PG) 32:1 and PG 34:1) were confirmed by accurate mass. **a** RP4 (25 C) and **b** R27.

We adopted the same approach to express and purify the RP4 pilus for structural studies as we reported for the H-pilus^13^. Briefly, RP4 pili were purified from *E. coli* MG1655 Δ*fimA* (lacking expression of type I pili) using the needle-shaving and precipitation method (see Methods section) (Fig. 1a). We then determined the cryo-EM structure of the RP4 pilus at 2.74 Å resolution (map:map FSC, 0.143 threshold) employing helical reconstruction in cryoSPARC by imposing helical (rise 15.15 Å, twist 24.62°) and C5 symmetry (Fig. 1b–d and Supplementary Fig. 1). The outer diameter of the pilus is ~80 Å and the lumen diameter ~20 Å, comparable to other conjugation pilus structures. The Coulomb map and structure confirm that the TrbC pilin is cyclized as a result of condensation of the Ser1 amine and Gly78 carboxyl residue and agree with the previous biochemical studies^16^ (Fig. 1c). The TrbC pilin contains three α-helices, α1-α3; the α1 and α2 helices are fused to form two α1/2 helices connected to a split α3 helix, α3_1_ and α3_2_, through a connecting loop. This arrangement resembles the conformation of the reported acyclic VirB2 pilin forming the T-pilus rather than the cyclic TrhA pilin forming the H pilus that has a split α3 helix connected via a loop to α1 and α2 helices (Fig. 2a); the TrbC and VirB2 pilin structures can be superimposed with an RMSD of 1.9 Å over 60 C_α_ atoms. Although the mature cyclic TrbC pilin has an additional 10 N-terminal residues compared to other pilins, the extra residues do not impact the overall structure. Unexpectedly, the structure revealed that the TrbC pilin is not associated with a phospholipid. Inspection of the maps did not reveal any lipid-like electron density in the proximity of the α2 or α3 helices where the bound lipids are usually located (Fig. 1c, e, and Supplementary Fig. 2). We validated that no phospholipid was present by mass spectrometry analysis on lipid extractions from the RP4 pilus (purified from cultures grown at either 25 °C or 37 °C), while PG was detected in extractions from the R27 pilus, which was used as a positive control (Fig. 3 and Supplementary Fig. 3). In particular, R27 derived pili were associated with PG species PG 32:1 and PG 34:1 similar to those previously observed^13^, while extract from RP4 pili showed a complete absence of lipids under two growth temperatures (Fig. 3 and Supplementary Fig. 3).

The lack of an associated phospholipid is unexpected as all pilus structures reported to date are associated with inner membrane lipids needed for biogenesis. In the lipidated pilins, the phospholipids provide additional contacts with neighboring pilin subunits and stability to the extensive intermolecular interactions (Fig. 2c and Supplementary Fig. 4). In contrast, interactions of the TrbC protomers are mediated primarily by intermolecular van der Waals forces (Fig. 2c). In the absence of a bound phospholipid at the protomer interface, the TrbC protomers come closer to ‘fill-in’ the space through a tight α3 and α2’ interface (Supplementary Fig. 5); this results in an offset of the two adjacent TrbC protomers by 24° rotation and 3 Å rigid body shift relative to the TrhA protomers (Fig. 2c and Supplementary Fig. 6). TrbC also makes extensive interactions with six surrounding subunits whereas in lipidated-pilus structures the associated phospholipids are found at the pilin interfaces (Fig. 2c). The presence of different phospholipid headgroups facing the lumen of pili change their overall charge from electropositive towards to neutral/slight electronegative (Fig. 1d, e). As the lumen of the lipid-free RP4 pilus is inherently negatively charged, this could potentially negate the need for a negative charge from the PG.

### An E. coli pgsA mutant can serve as a donor of RP4 but not R27

To confirm the dispensable role of glycerophospholipids in RP4 pili assembly and function, we utilized a viable *pgsA* null UE54 mutant^18–20^, completely lacking PG, as a donor. Conjugation of R27, R388, and pKpQIL was used as controls. The *pgsA* gene encodes phosphatidylglycerophosphate synthase, an enzyme that catalyzes the first committed step in the synthesis of PG and cardiolipin (CL). PG is a ubiquitous negatively charged phospholipid that is present in almost all bacteria and performs specific head group-specific ligand functions or reactions, serving as a biosynthetic precursor and is probably fundamental for terrestrial life^21^. Therefore, as with the other Δ*pgsA* strains, PG-less UE54 is viable only if it carries two additional gene mutations, including an *lpp* mutation necessary to prevent accumulation of the nascent major outer membrane lipoprotein (Lpp), which requires PG for its modification. Moreover, the Rcs phosphorelay system is constitutively activated in Δ*pgsA* mutants, resulting in cell lysis at 37 °C–42 °C, which is prevented by a Δ*rcsF* mutation^19,20^. The triple *pgsA lpp rcsF* mutant is viable at any temperature because it overcomes both the structural damage caused by the Lpp protein and the lethal Rcs temperature-sensitive stress response activated by the absence of PG in the *pgsA* mutant. Despite the major alterations in lipid composition UE54 cells divide and grow normally^22^.

**Fig. 4:**
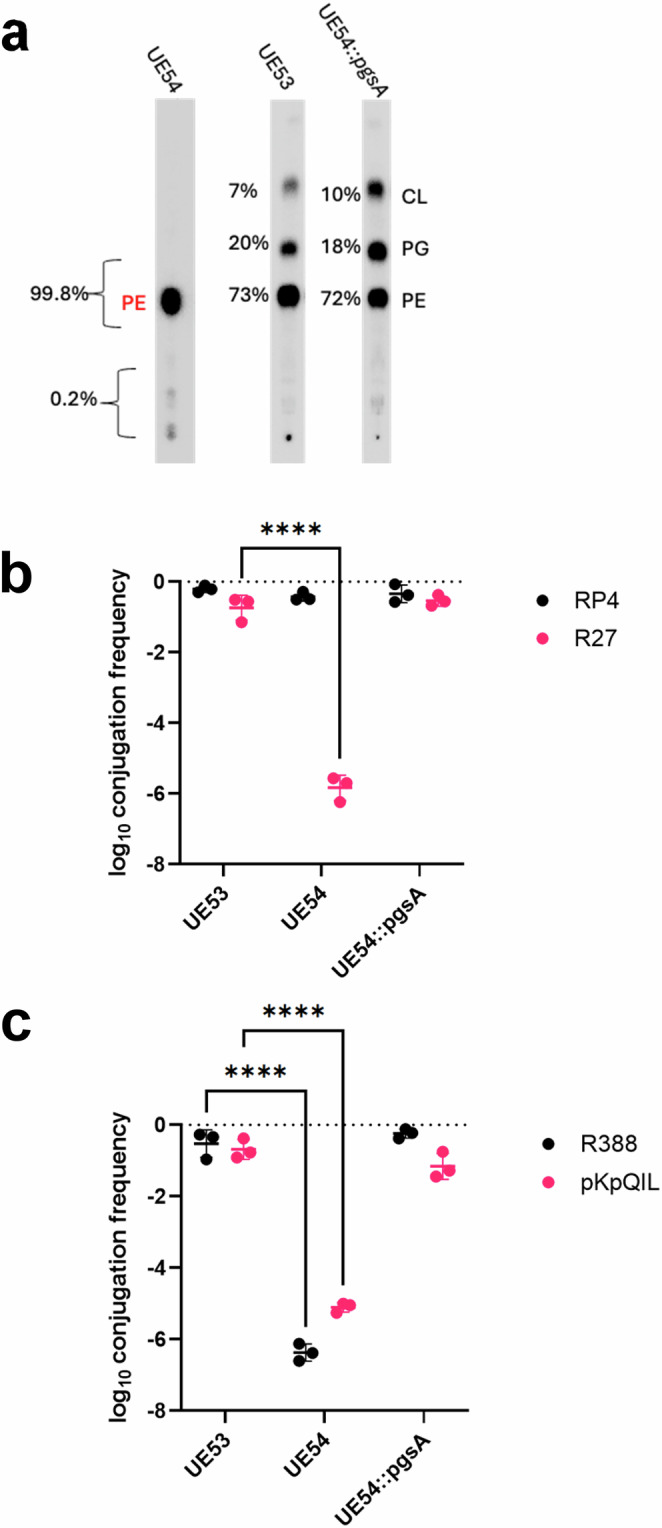
The role of PG in RP4 conjugation. **a** Lipid analysis profiles of wild type UE53 and UE54 *pgsA* null mutant *E. coli* with altered lipid composition. The UE54 (Δ*pgsA*) contains primarily PE and no detectable PG. Complementation of UE54 the with pPGL2134_pgsA plasmid restores the lipid composition to that seen in UE53. Uniformly [^32^P]PO_4_ labeled phospholipids (phosphatidylethanolamine (PE), phosphatidylglycerol (PG) and cardiolipin (CL)) were extracted and analyzed after separation by one-dimensional thin layer chromatography (TLC). **b** UE53 conjugates RP4 (black filled circles) and R27 (magenta filled circles) as efficiently into an *E. coli* MG1655 recipient, while UE54 conjugates RP4 but not R27. The R27 conjugation efficiency can be recovered in UE54 carrying the pPGL2134_pgsA plasmid. **c** R388 (black filled circles) and pKpQIL (magenta filled circles) display a similar PG dependency in UE54 that can be restored in UE54::*pgsA* carrying the pPGL2134_pgsA plasmid. Data are shown as mean ± standard deviation (*n* = 3 biological replicates). Conjugation frequency was analyzed by repeated-measures two-way ANOVA with Dunnett’s multiple-comparison test using UE53 as a control, **** *P* < 0.0001. Source data are provided as a Source Data file.

Although without PG and CL, the triple mutant can possess some anionic phospholipids, such as phosphatidic acid (PA) and N-acyl-phosphatidylethanolamine (N-acyl-PE)^22^, PA is accumulated only at trace amounts, and N-acyl-PE is not synthesized at all in logarithmically grown cells, and PE accounts for nearly 99.8% in *pgsA* null UE54 (Fig. 4A). [^32^P]PO_4_-labeling in conjunction with thin layer chromatography (TLC) demonstrates that the ∆pgsA mutant is producing only PE as the major zwitterionic phospholipid (Fig. 4a and Supplementary Fig. 7).

Following validation, we used the *∆pgsA* mutant and its isogenic wild-type strain UE53, as donors for RP4 and R27 and *E. coli* MG1655 as a recipient. While both RP4 and R27 were conjugated as efficiently from UE53, RP4 but not R27 was conjugated efficiently from the UE54 mutant (Fig. 4B). The fact that R27 plasmid cannot be conjugated efficiently when PG-less UE54 was used as a donor confirms that its pilus exhibits high selectivity for PG and the presence of PG is vital for conjugation. Moreover, PG is irreplaceable/non-interchangeable with other negatively charged lipids (PA and N-acyl PE) present in residual amounts in the *pgsA* null strain.

Due to the severely modified genotype of UE54, we performed a control experiment by complementing the mutant with plasmid-borne *pgsA*. The UE54 strain, which is already adapted to grow without *pgsA*, carries camR and kanR markers (UE54 lpp-2 _ara714 *rcsF*::mini-Tn10 cam *pgsA*::FRT-kan; genotype described in the Methods section). In this experiment, UE54 was transformed with the plasmid pPGL2134_pgsA_ampR, a high-copy plasmid constitutively expressing a *pgsA* gene^23^. As expected, the introduction of the plasmid completely restored the synthesis of PG (Fig. 4A, lane 3), effectively compensating for the *pgsA* deletion and rendering the strain independent of the *lpp* and *rcsF* bypass mutations. The conjugation efficiency of the R27 was restored when UE54::*pgsA* was used as the donor, further supporting the requirement of PG for R27 pilus biogenesis. We observed a similar behavior for R388 and pKpQIL pili, whose structures are also associated with PG lipids^7,8^. In these cases, pilus biogenesis is disrupted in ∆pgsA mutants and is restored upon introduction of the pPGL2134_pgsA_ampR plasmid.

The lipid-independent biogenesis of TrbC distinguishes this pilus from other conjugative pilus types. The cyclic pilins in both R27 and RP4 may contribute to pilus strength under the harsh environmental conditions. However, the presence or absence of a phospholipid is not a cyclic pilin feature as the R27 pilus incorporates phospholipids into its structure at a stoichiometric ratio. The RP4 plasmid’s TrbC protein is unique among cyclic pilins because it assembles into a functional pilus without requiring a phospholipid anchor. As such, the RP4 pilus provides the first known example of a lipid-independent mating pilus, expanding our understanding of the diverse mechanisms that bacteria employ to transfer genetic material.

The distinct differences between pilus structures in bacteria (cyclic and acyclic) and archaea^9,11^ give fundamental insights into plasmid biology, pilus biogenesis, DNA transfer, and evolution. It remains unclear why certain pili, acyclic or cyclic, such as the F- or H-pilus, adopt a 5-fold symmetry, whereas others, such as the pKpQIL pilus, adopt a one-start helical symmetry^7^; this plasticity in pilus architecture underscores the emergence of adaptable bacterial conjugation systems throughout evolution. Evidence for active retraction of the RP4 pilus remains limited compared with the F pilus, for which extension and retraction have been observed by live-cell imaging^24–27^. However, the mechanism of pilus retraction is still not well understood, and proposed roles for phospholipids remain largely hypothetical. In retractile systems, such as the F pilus, phospholipids have been suggested to facilitate pilin assembly and disassembly, contribute to filament flexibility, and influence relaxase-ssDNA transfer^4^. By contrast, for the lipidated R388 pilus, it has been proposed that the mode of phospholipid binding may disfavor retraction^8^. At present, there is little direct evidence that lipids are strictly required for pilus retraction. The absence of lipid-like electron density in the TrbC maps highlights a fundamental difference in the assembly and structure of the conjugative apparatus encoded by IncP plasmids, which are found in diverse bacterial species (Supplementary Fig. 8). The absence of bound phospholipids in RP4 is not consistent with current models proposing a central role for phospholipids in pilus biogenesis and function. Our data suggest that bound phospholipids are not required for TrbC extraction from the inner membrane, pilus assembly, and integrity in RP4. In addition, the efficient conjugation frequencies we observe are not consistent with models in which negatively charged phospholipid headgroups within the pilus lumen are essential for relaxase-ssDNA transfer. Taken together, our findings suggest that, at least for RP4, phospholipids are not required for pilus assembly or DNA transfer.

## Methods

### Expression and purification of RP4 pilus

The RP4 pilus was purified following the protocol used to prepare the H pilus^13^. Briefly, bacterial cells from an overnight culture were sheared through 25 G needles 25–30 times and were centrifuged at 50,000 xg for 1 h. The pilus filaments were precipitated using PEG 6000 at a final concentration of 5% with stirring for 1 h, which was followed by centrifugation at 50,000 x g for 30 min. The supernatant was removed, and the pellet was resuspended in 200 μL imaging buffer (50 mM Tris-HCL pH 8, 200 mM NaCl). The solution containing the filaments was dialyzed overnight in imaging buffer. Sample purity was judged by SDS-PAGE.

### Grid preparation and cryo-EM data collection

For grid preparation, 3 µL of the purified sample at ~4 mg/mL was applied to a glow-discharged carbon grid (Quantifoil R1.2/1.3, Cu, 300 mesh). The grid was blotted with a blotting force of 0 for 3 s at 4 °C and 100% humidity, and flash-frozen in liquid ethane using a Vitrobot Mark IV (Thermo Fisher Scientific). Cryo-EM data were collected on 300 kV Titan Krios G2 (Thermo Fisher Scientific) with a Falcon 4i detector and Selectris X energy filter at the Electron Bioimaging Center (eBIC), UK. A total of 3,405 movies were collected at a magnification of ×130,000 (pixel size of 0.921 Å/pixel) with a total electron dose of 50 e/Å2. The defocus range was set between −0.8 and −1.8 µm. All data were automatically acquired using EPU software. The data collection parameters are summarized in Supplementary Table 1.

### Cryo-EM data processing and model building

The data were processed using cryoSPARC (v.4.6.1)^28^. Data collected from a Titan Glacios and Krios were analyzed separately, 2D images and initial volume from the Glacios dataset were used for initial reconstruction. Both datasets were processed following similar steps. Motion correction was performed using Patch Motion Correction, and CTF estimation for micrographs was done by Patch CTF estimation^28^. Particles from the Glacios dataset were manually picked from all micrographs and extracted in a binned state at 2.76 Å/pix. The extracted particles were classified in two rounds in 2D classification, and selected high-quality particles were used as references for the Filament tracer. Three rounds of 2D classification from the whole dataset was used to select particles for helical reconstruction. Helical symmetry search job suggested an axial rise of ~15.3 Å and a twist of ~24.6⁰, along with 5-fold point group symmetry. These symmetry parameters were used as starting parameters for helical reconstruction using Helix refine. A 4.0 Å RP4-pilus map was reconstructed from the Glacios data set. The 2D references and 3D model from the Glacios dataset were used as reference for the Krios dataset. The Krios dataset consisted of 3405 movies. After Motion Correction and CTF refinement, template pick was performed with Filament tracer using a 2D reference from the Glacios dataset. 4,302,764 particles were extracted in the binning state. Three rounds of 2D classification were performed, and 10,792 particles were selected. Using 3D volume and helical parameters from the Glacios dataset, Helical refinement, local CTF refinement and reference-based motion correction were performed several times. The final RP4 pilus helical reconstruction had a 2.74 Å resolution, with a rise of 15.15 Å and a twist of 24.62°.

Ab initio model building and real space refinement were performed in PHENIX (2.0)^29,30^. The model was subjected to iterative cycles of refinement and manual rebuilding in COOT^31^. The structural model was validated using MolProbity^32^. The processing parameters and refinement statistics are summarized in Supplementary Table 1.

### Determination of steady-state phospholipid composition by radiolabeling

To determine the steady-state phospholipid composition by radiolabeling, cells were uniformly labeled with 5 µCi/ml of [^32^P]PO_4_ after dilution to OD_600_ of 0.05 to initiate logarithmic growth after dilution of the overnight culture. Cells were harvested by centrifugation, and phospholipids were extracted by the acidic Bligh-Dyer procedure. After labeling, cells were harvested by centrifugation and resuspended in 0.2 mL of acidic solution (0.5 M NaCl in 0.1 N HCl). Lipids were extracted by adding 0.6 mL of solution 2 (chloroform:methanol, 1:2, v/v), followed by vortexing for 30 minutes. Subsequently, 0.2 mL of resuspension buffer was added, and the sample was vortexed for an additional 10 minutes. Phase separation was achieved by centrifugation at 13,000 × *g* for 5 min. The lower chloroform phase, containing the extracted phospholipids, was collected. An aliquot corresponding to approximately 1,000–2,000 counts per minute (cpm) of radiolabeled phospholipid was used for thin-layer chromatography (TLC) analysis. One-dimensional TLC was performed to resolve individual phospholipid species. Boric acid-impregnated Merck Kieselgel 60 TLC plates (0.25 mm thickness) (EMD, Gibbstown, NJ) were activated and developed with a solvent consisting of chloroform:methanol:acetic acid (65:25:8 v/v). The spots were assigned to different *E. coli* phospholipids based on TLC mobility (Rf values) of in-house standards and utilization of different lipid mutants, including viable *pssA* null and therefore not synthesizing major phospholipid PE. Radiolabeled lipids were detected using a Typhoon FLA 9500 PhosphoImager (GE) and quantified with ImageQuant™ software. Stored images were processed and quantified using ImageQuantTM software. Phospholipid content is expressed as mol% of total phospholipid based on the intensity of the captured signal on the Phosphor screen generated by the radiolabeled spots on the TLC plate. The results presented are representative of three or more determinations. All TLC analyses were performed in at least two independent experiments to ensure reproducibility.

### Lipid analysis of Pili by mass spectrometry

Purified pili were subjected to lipid extractions as previously described^4^. Briefly purified pili (~0.05 mg) were treated with phospholipase A2 (0.1 units) in PBS for 16 h at 37 °C, followed by heat inactivation. The remaining Bligh and Dyer extracted lipids were analyzed with a Thermo Exactive Orbitrap mass spectrometer in both positive and negative ion modes.

### Bacterial strains, growth conditions, and conjugation

UE54 (*lpp*-2 Δ*ara714 rcsF*::mini-*Tn10 cam pgsA*::FRT-*kan*-FRT) is a null for the *pgsA* gene and is therefore unable to synthesize phosphatidylglycerol (PG). UE53 (MG1655 *lpp*-2 Δ*ara714 rcsF*::mini-*Tn10* cam) was utilized as the parental strain with wild-type phospholipid composition. Cells were grown in LB medium containing 10 g/L Bacto-tryptone, 5 g/L Bacto-yeast extract, and 10 g/L NaCl.

UE53 or UE54 containing the derepressed R27, RP4, R388, or pKpQIL were used as donors; the R27 and RP4 plasmids encode chloramphenicol resistance, and the R388 and pKpQIL encode trimethoprim and ertapenem resistance, respectively. Streptomycin-resistant *E. coli* MG1655 was used as a recipient. The donor and recipients were grown in LB at 37 °C overnight, mixed in a ratio of 10:1 (R27), 1:1 (RP4), 1:1 (R388) and 8:1 (pKpQIL), diluted 1 in 25 in PBS, and 40 μL spotted onto LB agar plates (no selection); these ratios were previously shown to result in the highest conjugation frequency^33–36^. Following incubation at 37 °C for 3 h to recover, R27 conjugation was allowed to proceed overnight at 25 °C. For RP4, R388, and pKpQIL conjugation, the mixture was incubated overnight at 30 °C. The conjugation mixtures were collected and resuspended in 1 mL of PBS following serial dilution from 10^−1^ to 10^−8^. For selection of transconjugants, the serial dilutions were plated on LB containing either 30 µg/mL chloramphenicol (R27 and RP4) or 50 µg/mL trimethoprim (R388) or 0.25 µg/mL ertapenem (pKpQIL) and 50 µg/mL streptomycin and incubated at 37 °C overnight. Data are presented as proportions of transconjugants/recipients. Conjugation frequency was compared by two-way ANOVA followed by Dunnett’s multiple-comparison test in GraphPad Prism (v.10).

### UE54 complementation

Strains UE54 and JA200 (pPGL2134_pgsA_ampR)^23,37^ were prepared as overnight cultures at 37 °C. Plasmid pPGL2134 was extracted by using Plasmid Mini-Prep Kit (NEB). UE54 strain was diluted in 1:100 for the room-temperature competent cells preparation by placed onto the Thermo shaker 37 °C 900 rpm for 2.5 h. The pPGL2134 was transformed into UE54 by using electroporation under 2.5 kV (Bio-Rad). The cells were plated on an ampicillin-resistant LB agar plate after 1.5 h recovery. The complemented strain was used for conjugation assays.

The plasmids and strains used in this study are shown in Supplementary Table 2.

### Reporting summary

Further information on research design is available in the Nature Portfolio Reporting Summary linked to this article.

## Supplementary information

Supplementary Information

Reporting Summary

Transparent Peer Review file

## Source data

Source data

## Data Availability

The cryo-EM maps have been deposited in the Electron Microscopy Data Bank (EMDB) under accession code EMD-65024. The structural coordinates have been deposited to the RCSB Protein Data Bank (PDB) under the accession code 9VF4. Raw images were submitted to the Electron Microscopy Public Image Archive (EMPIAR) with ID EMPIAR-12839. The previously determined pilus structures used in this study are available through the PDB under the accession codes 5LEG and 9MOQ (F), 9HVC (H), 8EXH (T), 8CW4 (N) and 8S6H (W). The source data underlying Fig. 4 are provided as a Source Data file. Source data are provided with this paper.
