# Supplementary material for: Phospholipid-independent biogenesis and function of the RP4 conjugation pilus
Source: Nat Commun. 2026 Jun 17;17:7671. doi: 10.1038/s41467-026-74409-x (PMC13434634; doi:10.1038/s41467-026-74409-x)
Supplement: Supplementary file 1 [file 41467_2026_74409_MOESM1_ESM.pdf]

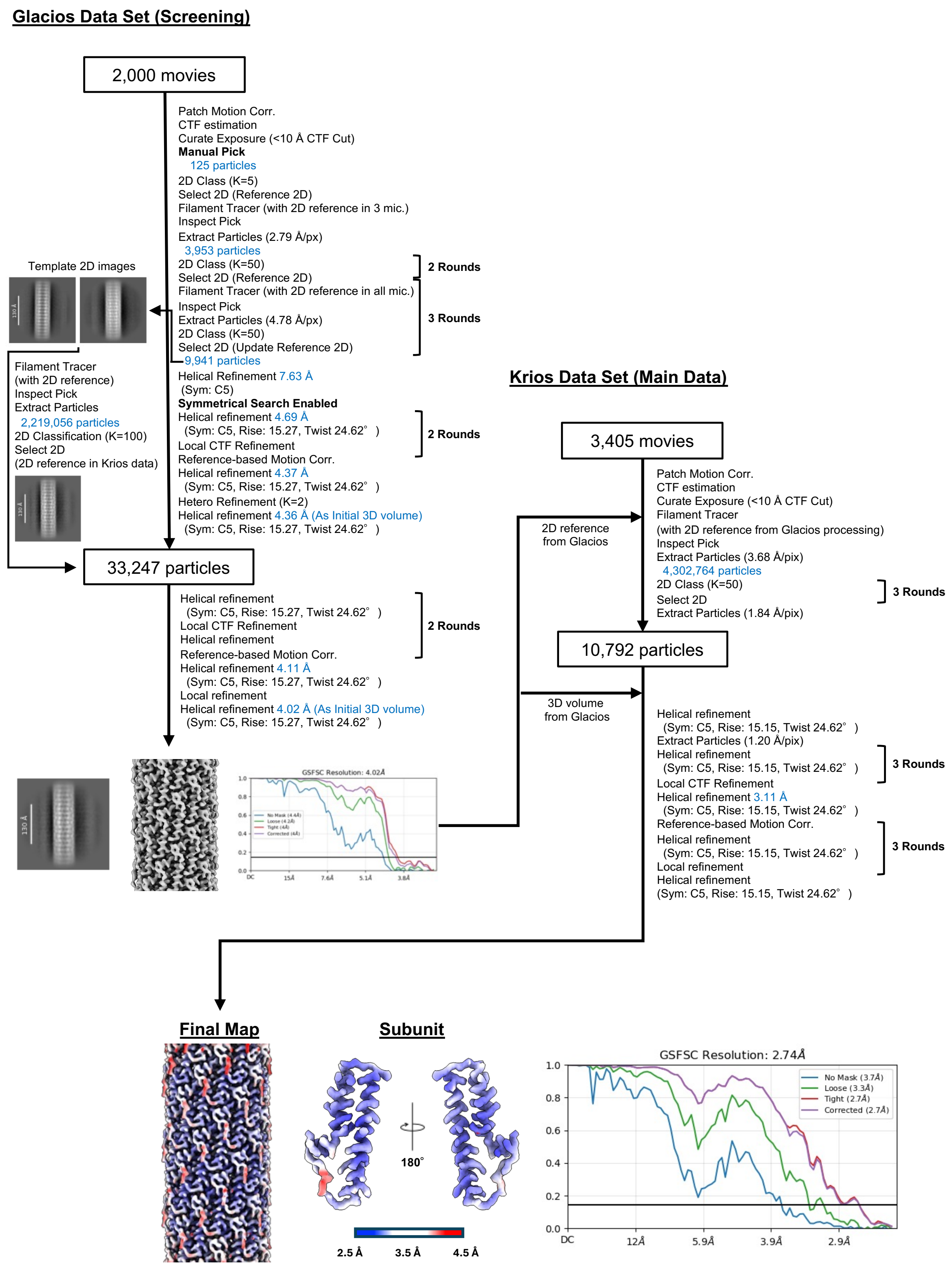

Supplementary Figure 1. Cryo-EM data processing. All the processing was performed in cryoSPARC (v.4.6.1). The final map was colored according to the local resolution. Gold-standard FSC curve of the final map was shown. The resolution was cut-off at FSC=0.143.

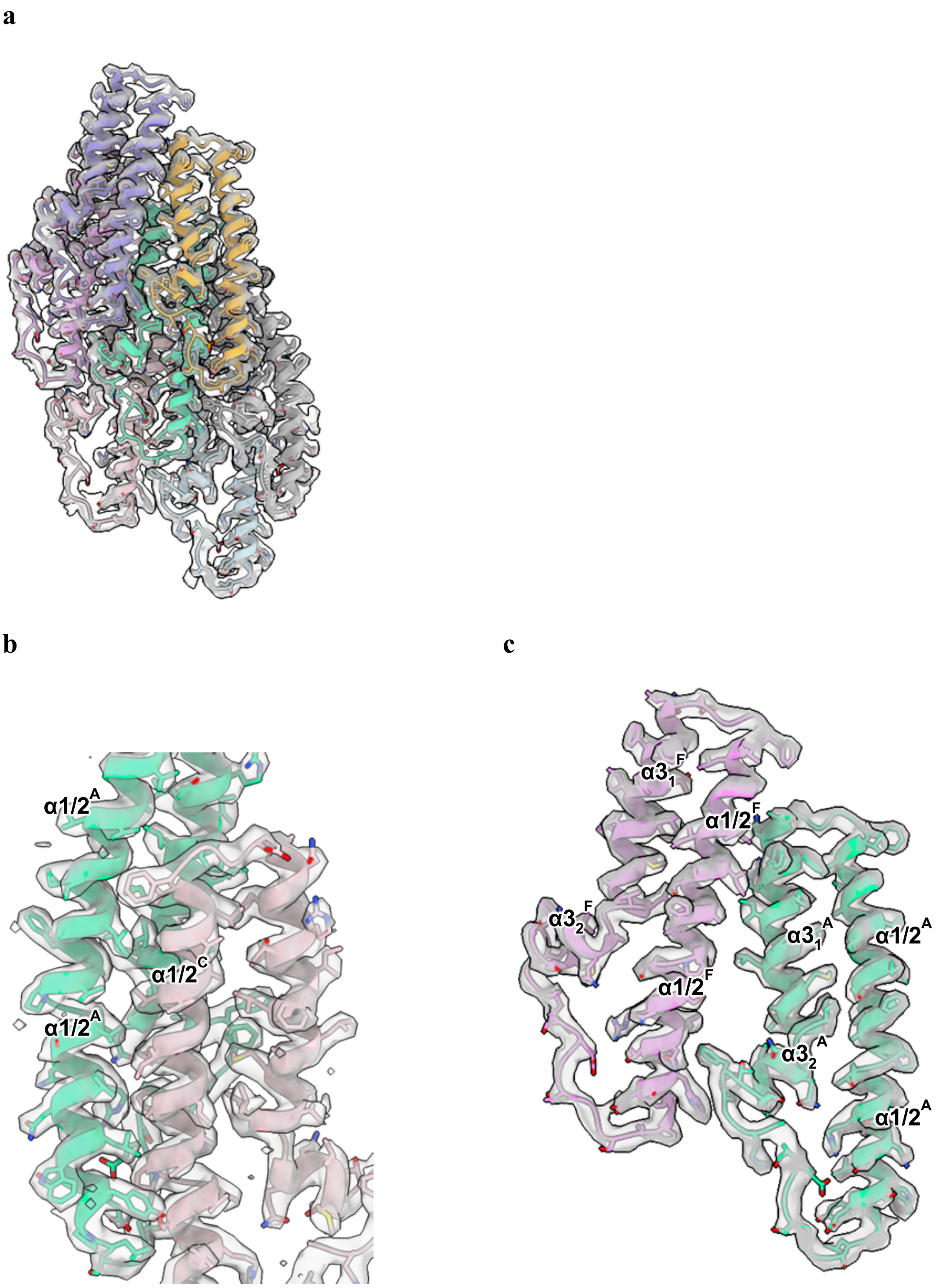

Supplementary Figure 2. Cryo-EM maps along the TrbC pilin interface. (a) Coulomb density extended to cover several TrbC pilin subunits. (b and c) Close up inspection of te different neighbouring TrbC pilin subunits does not reveal any lipid density.

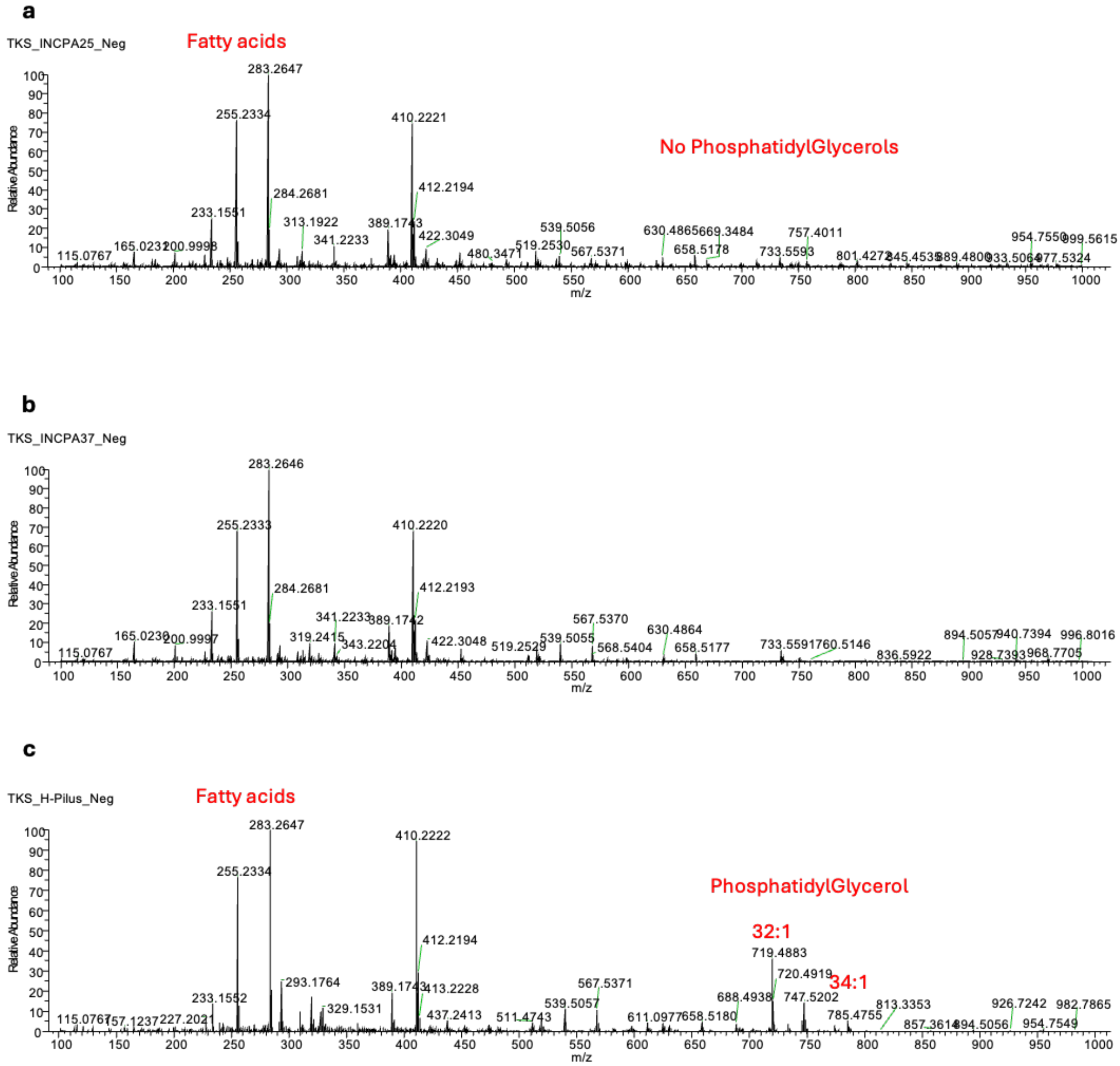

Supplementary Figure 3. Mass spectrometric analysis of lipids associated with purified pili. Negative ion mode survey scans (100-1000 m/z) of lipids isolated from pili after treatment with phospholipase A2. Annotated phospholipid identities were confirmed by accurate mass. (a) RP4 (25C); (b) RP4 (37C) and (c) R27

a

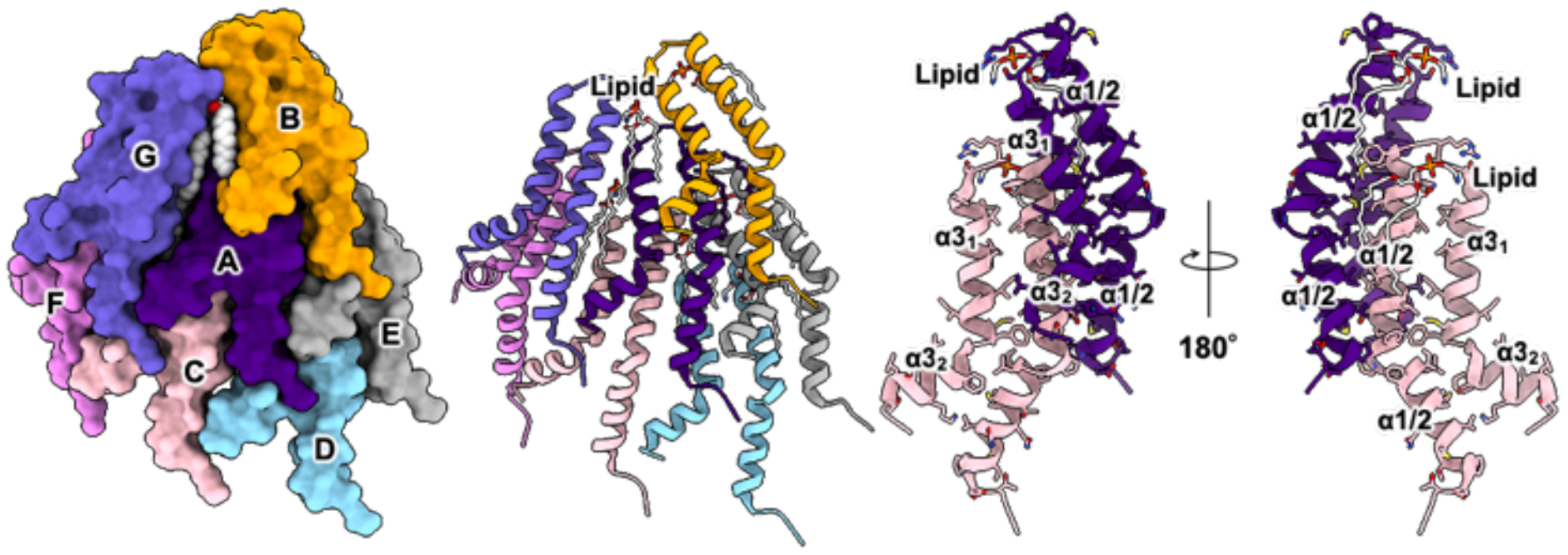

b

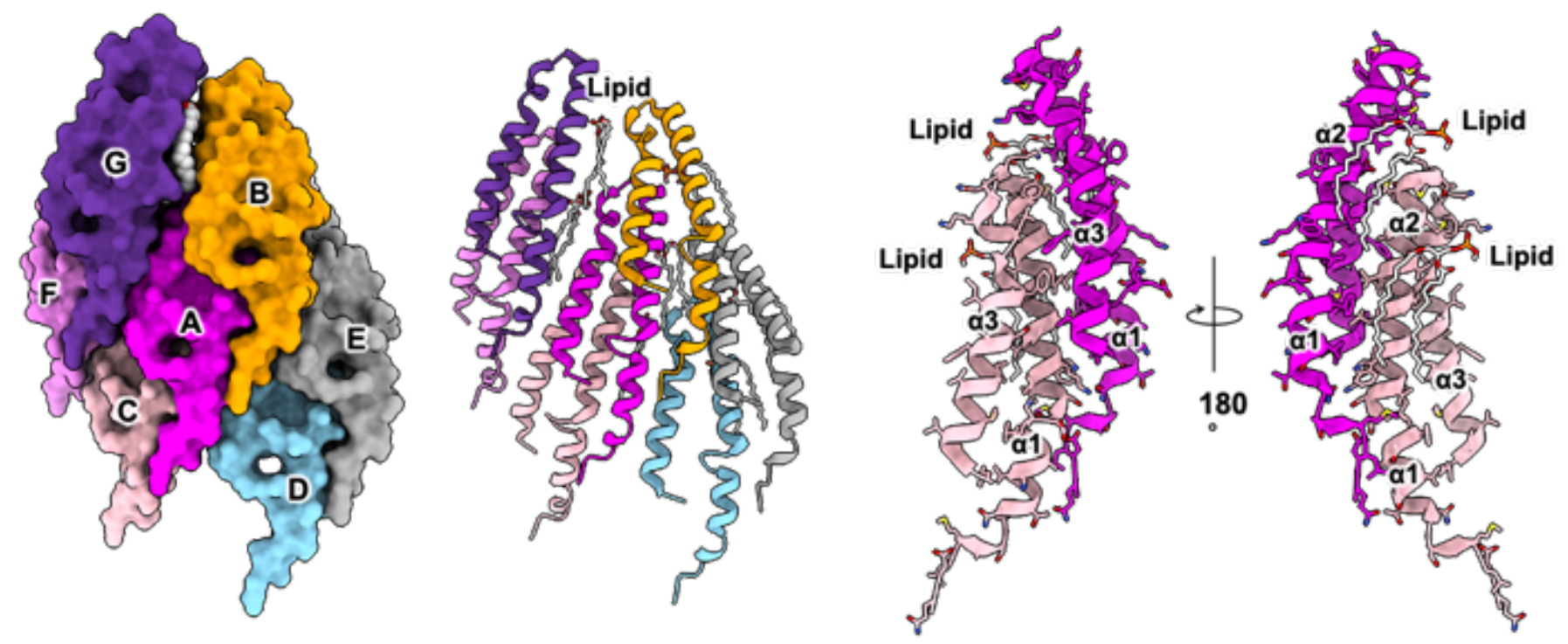

Supplementary Figure 4. Protomer interface of pilins from different pilus. (a) T pilus (PDB ID: 8EXH) and (b) F pilus (PDB ID: 9MOQ)

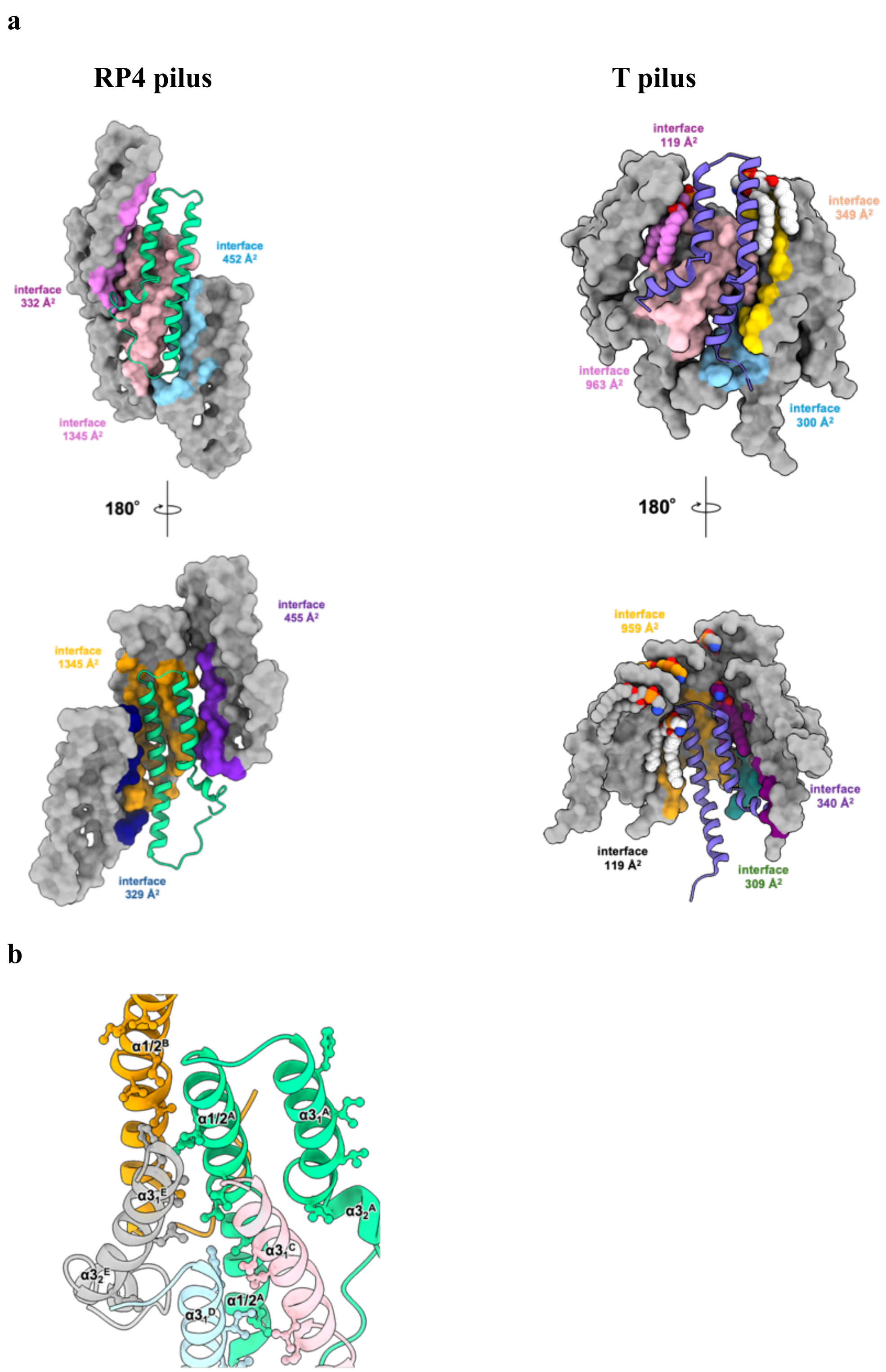

Supplementary Figure 5. TrbC pilin intrasubunit buried interface. (a) The TrbC buried interface with adjustment protomers is more extensive compared to the VirB2 pilins. The PG lipids have been included in the calculations. (b) The residues involved in the TrbC interface are shown as sticks.

a

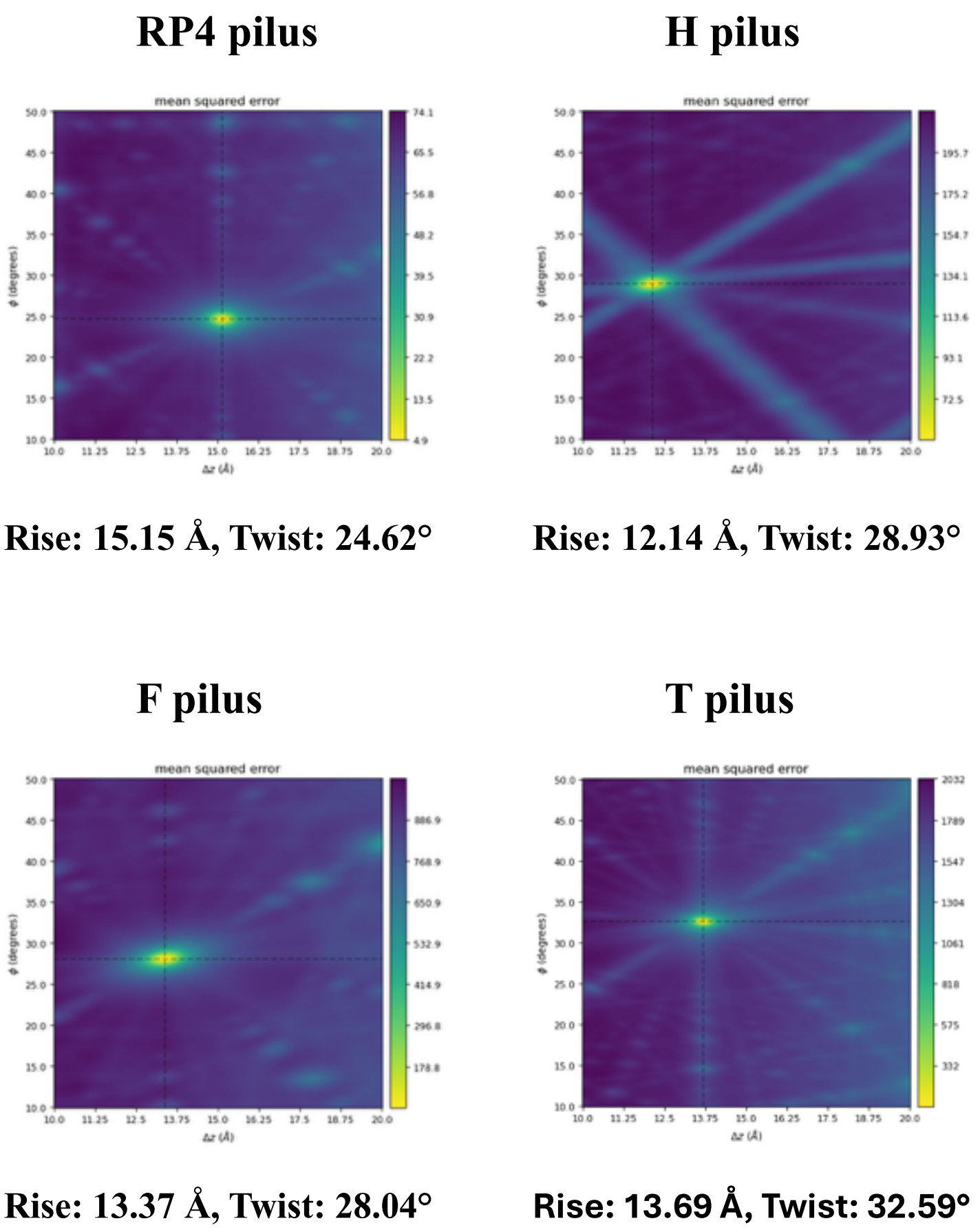

b

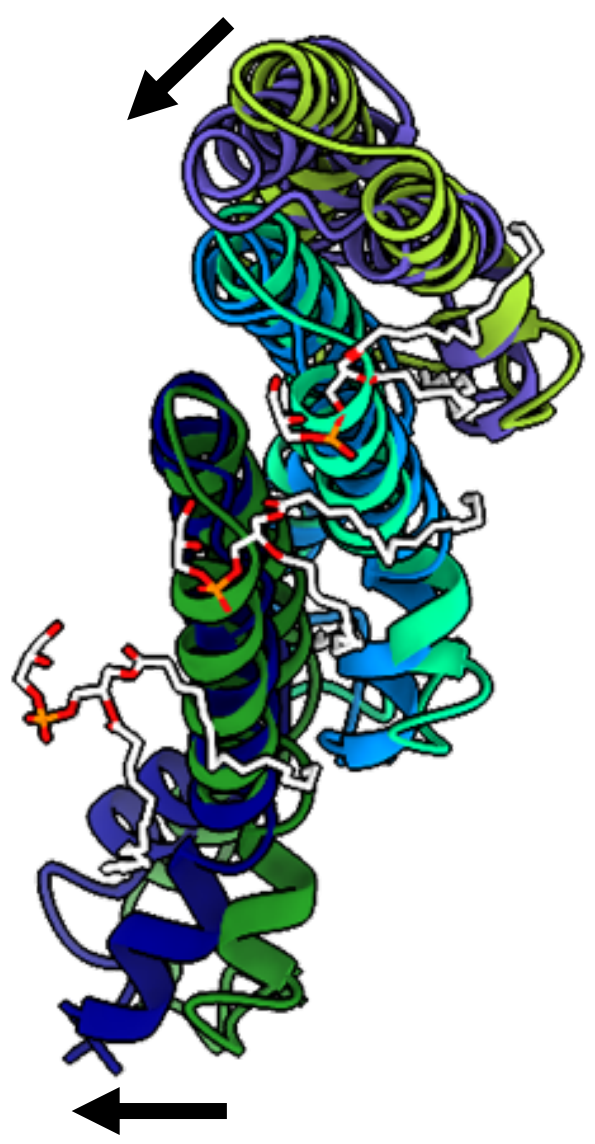

Supplementary Figure 6. The lack of lipids in TrbC alters the pilin interface. (a) Symmetry search calculation of the rise and twist between different pili. (b) Three subunits of the RP4

pilus (shades of green) and H pilus with PG (shades of blue and white sticks) have been superimposed onto the middle subunit. The adjustment subunits display a shift and twist as calculated in panel (a).

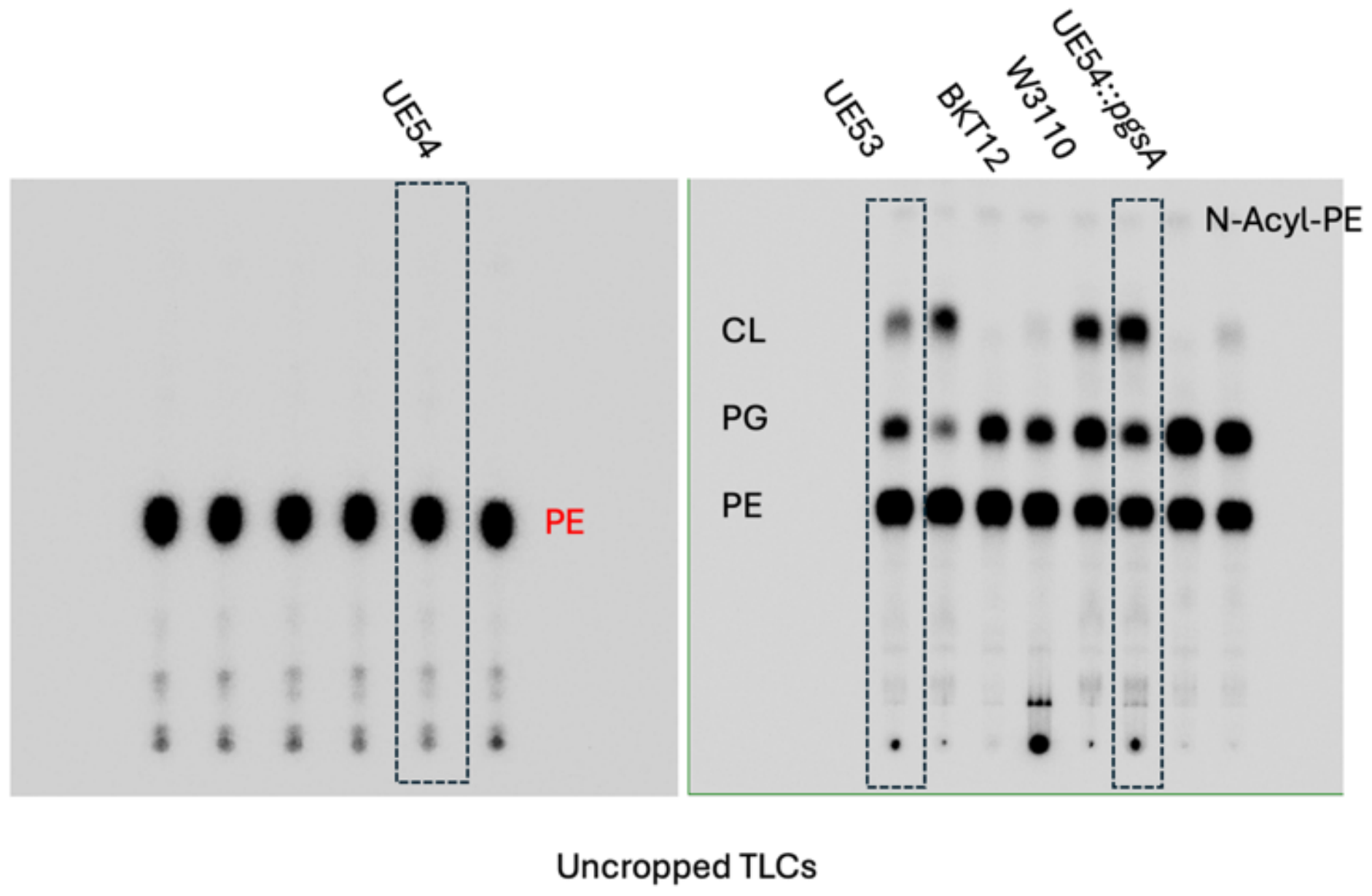

Supplementary Figure 7. Uncropped gels from Figure 4. Dashed lines indicate the lanes used in Figure 4.

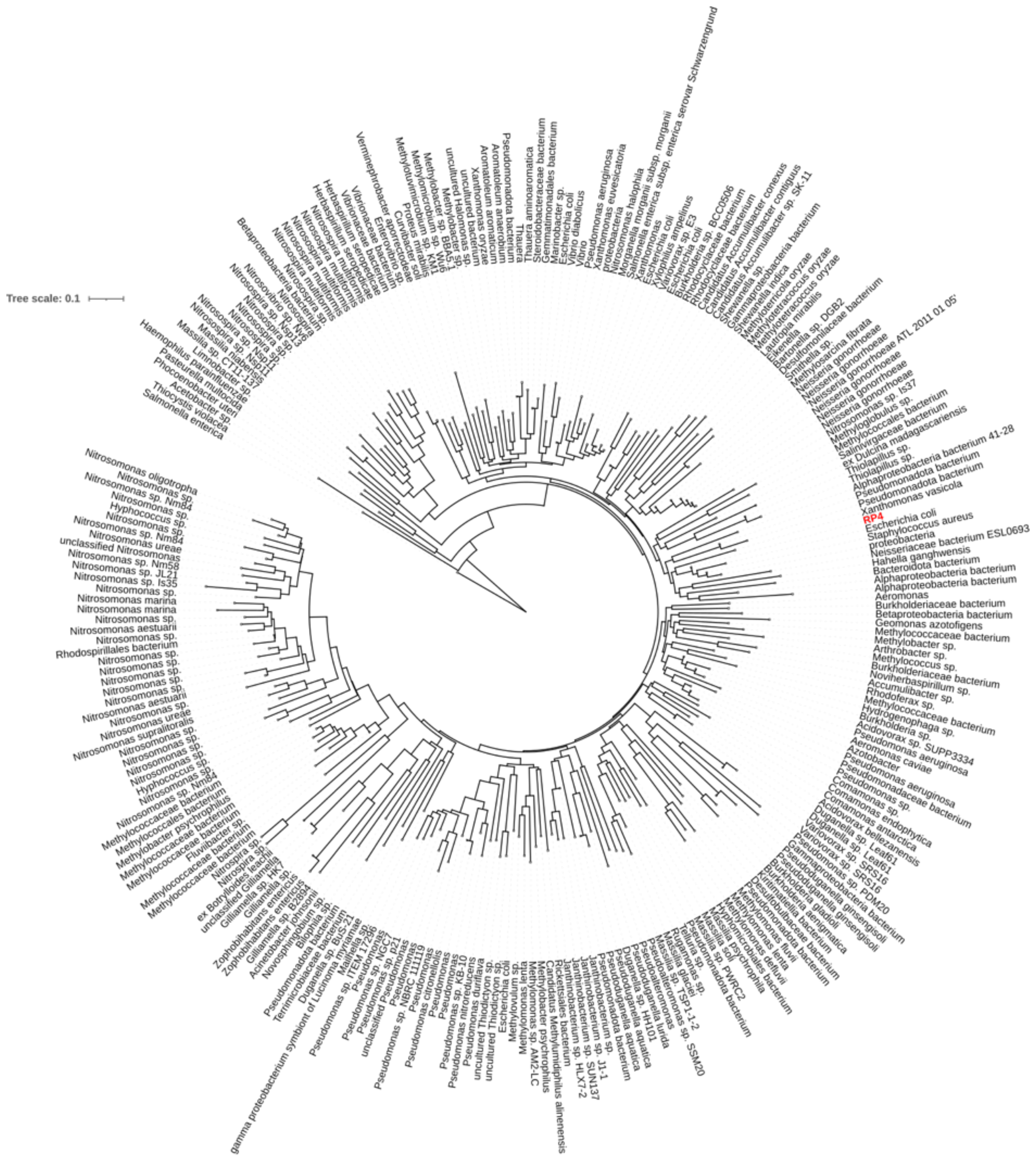

Supplementary Figure 8. Phylogenetic analysis of TrbC pilin. Circular phylogenetic tree at level of bacterial species. The tree was generated with NCBI BLASTP and viewed in ITOL.

Supplementary Table 1. Cryo-EM data collection and refinement statistics

	RP4-pilus
**PDB entry**	9VF4
**EMDB entry**	EMD-65024
**Data collection and processing**	
Magnification	130,000
Microscope	Titan Krios
Voltage (kV)	300
Detector	Falcon 4i
Electric exposure (e^-/Å)	50
Deforcus range (μm)	-0.8 to -1.8
Pixel size (Å)	0.921
Data Processing Program	cryoSPARC (v.4.6.1)
Movies	3,405
Initial / Final particle images (no	4,302,764 / 10,792
Symmetry imposed	C5
Helical rise (Å)	15.15
Helical twist (°)	24.62
Map resolution (Å)	2.75
FSC threshold	0.143
**Refinement**	
Refinement Program	PHENIX (v.1.20.1)
Model resolution (Å)	
FSC threshold = 0.143	2.73
Model composition	
Non-hydrogen atoms	574
Protein residues	78
R.m.s. deviations	
Bond length (Å)	0.006
Bond angles (°)	0.503
Validation	
MolProbity score	1.38
Clashscore	4.29
Ramachandran plot	
Favored / Allowed (%)	98.68 / 1.32
Disallowed (%)	0.00
Mask CC	0.86

Supplementary Table 2. Plasmids and strains used in this study.

**Plasmids**	**Description**	**Source and References**
RP4	Prototype IncP plasmid	Prof. Christopher Thomas[1]
R27	Prototype derepressed IncH plasmid	Prof. Carlos Balsalobre[2]
R388	Prototype IncW plasmid	Prof. Gabriel Waksman[3]
pKpQIL	KPC-2-encoding IncFIIK plasmid	Lab collection[4]
pPGL2134_pgsA_ampR	Derivative of pSC101 constitutively expressing *pgsA*	Lab collection (Prof. Bogdanov)[5]

**Strains**	**Description**	**Source and References**
UE53	MG1655 *lpp*-2 Δ*ara714 rcsF*::mini-*Tn10* cam	Lab collection (Prof. Bogdanov)[6]
UE54	*lpp*-2 Δ*ara714 rcsF*::mini-*Tn10 cam pgsA*::FRT-*kan*-FRT	Lab collection (Prof. Bogdanov)[6]
E. coli MG1655 streptomycin	E. Coli K12 strain harbours the pACBSR plasmid that confers resistance to streptomycin	Lab collection
JA200	*thr-1, araC14, leuB6(Am), lacY1, glnX44(AS), galK2(Oc), galT22, λ-, ΔtrpE63, recA56, xylA5, mtl-1, thiE1*	Lab collection (Prof. Bogdanov)[7]

Supplementary References

1 Figurski, D. H. & Helinski, D. R. Replication of an origin-containing derivative of plasmid RK2 dependent on a plasmid function provided in trans. *Proc Natl Acad Sci U S A* **76**, 1648-1652 (1979). https://doi.org/10.1073/pnas.76.4.1648

2 Gibert, M., Paytubi, S., Madrid, C. & Balsalobre, C. Temperature Dependent Control of the R27 Conjugative Plasmid Genes. *Front Mol Biosci* **7**, 124 (2020). https://doi.org/10.3389/fmolb.2020.00124

3 Vadakkepat, A. K. *et al.* Cryo-EM structure of the R388 plasmid conjugative pilus reveals a helical polymer characterized by an unusual pilin/phospholipid binary complex. *Structure* **32**, 1335-1347 e1335 (2024). https://doi.org/10.1016/j.str.2024.06.009

4 Low, W. W. *et al.* Mating pair stabilization mediates bacterial conjugation species specificity. *Nat Microbiol* **7**, 1016-1027 (2022). https://doi.org/10.1038/s41564-022-01146-4

5 Gopalakrishnan, A. S., Chen, Y. C., Temkin, M. & Dowhan, W. Structure and expression of the gene locus encoding the phosphatidylglycerophosphate synthase of Escherichia coli. *J Biol Chem* **261**, 1329-1338 (1986).

6 Mileykovskaya, E. *et al.* Phosphatidic acid and N-acylphosphatidylethanolamine form membrane domains in Escherichia coli mutant lacking cardiolipin and phosphatidylglycerol. *J Biol Chem* **284**, 2990-3000 (2009). https://doi.org/10.1074/jbc.M805189200

7 Ohta, A., Waggoner, K., Radominska-Pyrek, A. & Dowhan, W. Cloning of genes involved in membrane lipid synthesis: effects of amplification of phosphatidylglycerophosphate synthase in Escherichia coli. *J Bacteriol* **147**, 552-562 (1981). https://doi.org/10.1128/jb.147.2.552-562.1981
