# Supplementary material for: Phospholipid-independent biogenesis and function of the RP4 conjugation pilus
Source: Nat Commun. 2026 Jun 17;17:7671. doi: 10.1038/s41467-026-74409-x (PMC13434634; doi:10.1038/s41467-026-74409-x)
Supplement: Supplementary file 3 [file 41467_2026_74409_MOESM3_ESM.pdf]

nature portfolio

Peer Review File

Phospholipid-independent biogenesis and function of the RP4 conjugation pilus

Corresponding Author: Dr Konstantinos Beis

This manuscript has been previously reviewed at another journal. This document only contains information relating to versions considered at Nature Communications.

This file contains all reviewer reports in order by version, followed by all author rebuttals in order by version.

Version 0:

Reviewer comments:

Reviewer #2

(Remarks to the Author)
Ishimoto et al report structural, biochemical and biological evidence that the RP4 conjugative pilus is assembled and functional in the absence of associated lipids, which distinguishes it from other previously characterized conjugation systems. This is an unexpected result which contributes to our understanding of the variability of conjugative machineries. The manuscript is an improved version of an original submission to [REDACTED] in which the authors successfully address most of the reviewers' concerns. The results are sound and clear-cut. However, there are two issues which still require their attention / further work:
1. 3 out of 4 reviewers claimed that their statement that the absence of lipids explained the rigidity and lack of retractability of the RP4 pilus was too strong. Overall, previous evidence links lipid composition with F pilus retractability; and there is a proposed explanation to why the R388 pilus would have lipids, yet it would be non-retractable. But neither of these previous results, nor the present ones, justify the claim that absence of lipids is the cause of RP4 non-retractability, less to say, rigidity. Even though the authors, in their response to the reviewers, insist on their claim, the manuscript does not state it any longer. Instead, the last paragraphs of the MS present current evidence relating lipids and retractability in F and R388. However, this last part of the text requires rewriting because it is difficult to follow and the message is not clear.

2. Conjugation results are an essential part of the work, and the results shown in Fig 4 are clear-cut and exciting. However, the corresponding Materials and Methods section is really poor and needs profound changes:

a. A supplementary Table listing strains and plasmids should be included, listing their source. They mention a JA200 and a recipient MG1655 Sm-resistant strain, where did it come from, which is its genotype? What is plasmid pPGL2134_pgsA_ampR like? They mention in the results that donor strains harbored the "derepressed" RP4 and R27 plasmids, are they using the natural isolates or some mutants? And what about R388 or pKpQIL?
b. The conjugation protocol is really odd: 3 hours at 37ºC, then o/n incubation at 25ºC for R27, and 30ºC for RP4. It is particularly surprising since 37ºC is a restrictive temperature for R27, so this plasmid should not be mating under these circumstances. In addition, the donor:recipient ratio is 10 times lower for RP4. What is the rationale for all this?
c. The authors include the results of conjugations with R388 and pKpQIL in Figure 4, but there is no mention to conjugation conditions in MM section (they do not even mention which antibiotic resistance they are using to select them). If RP4 is assayed in different conditions to the other 3 plasmids (with respect to temperature, time of incubation, and especially, donor:recipient ratio), extra controls should be run to discard that the differences in conjugation frequencies are caused by any of these experimental conditions.
d. One reviewer sensibly asked for the CFU data and authors claimed to have included them in the SI, but they are not visible anywhere. This is a relevant information, please do show those data.

Reviewer #3

(Remarks to the Author)

The authors have adequately addressed the comments provided.

Reviewer #4

(Remarks to the Author)
The revised manuscript by Ishimoto et al. presents additional data supporting the major conclusions. Overall, the manuscript provides novel insights into conjugative pilus biology and function, and presents the first structure of a lipid-independent mating pilus. In response to prior review, the authors have satisfactorily addressed my major concerns with one caveat - based on the presented data, the speculative statement asserting that the absence of phospholipids imparts RP4 pilus rigidity and prevents pilus retraction remains questionable. This point was also raised by Reviewer 2 in the prior review. While the authors provide arguments supporting their assertion, and the new structural information suggests one plausible mechanism that may account for for pilus rigidity and inability to retract, the language in the manuscript should be significantly modified to reflect alternative mechanistic possibilities.

Open Access This Peer Review File is licensed under a Creative Commons Attribution 4.0 International License, which permits use, sharing, adaptation, distribution and reproduction in any medium or format, as long as you give appropriate credit to the original author(s) and the source, provide a link to the Creative Commons license, and indicate if changes were made.
In cases where reviewers are anonymous, credit should be given to 'Anonymous Referee' and the source.
The images or other third party material in this Peer Review File are included in the article’s Creative Commons license, unless indicated otherwise in a credit line to the material. If material is not included in the article’s Creative Commons license and your intended use is not permitted by statutory regulation or exceeds the permitted use, you will need to obtain permission directly from the copyright holder.
To view a copy of this license, visit https://creativecommons.org/licenses/by/4.0/

Ishimoto et al report structural, biochemical and biological evidence that the RP4 conjugative pilus is assembled and functional in the absence of associated lipids, which distinguishes it from other previously characterized conjugation systems. This is an unexpected result which contributes to our understanding of the variability of conjugative machineries.

The manuscript is an improved version of an original submission to [REDACTED] in which the authors successfully address most of the reviewers' concerns. The results are sound and clear-cut. However, there are two issues which still require their attention / further work:

1. 3 out of 4 reviewers claimed that their statement that the absence of lipids explained the rigidity and lack of retractability of the RP4 pilus was too strong. Overall, previous evidence links lipid composition with F pilus retractability; and there is a proposed explanation to why the R388 pilus would have lipids, yet it would be non-retractable. But neither of these previous results, nor the present ones, justify the claim that absence of lipids is the cause of RP4 non-retractability, less to say, rigidity. Even though the authors, in their response to the reviewers, insist on their claim, the manuscript does not state it any longer. Instead, the last paragraphs of the MS present current evidence relating lipids and retractability in F and R388. However, this last part of the text requires rewriting because it is difficult to follow and the message is not clear.

Reviewer #4 raised a similar point. We have tuned down the claims and revised the manuscript accordingly (changes are highlighted in yellow). We are presenting the RP4 structure as an alternative model for pilus assembly and function, without making any claims of retraction or rigidity.

2. Conjugation results are an essential part of the work, and the results shown in Fig 4 are clear-cut and exciting. However, the corresponding Materials and Methods section is really poor and needs profound changes:

We have updated our Materials and Methods to include all the plasmids and conditions (ratio of donor:recipient) and antibiotics used. We have included appropriate references as well.

a. A supplementary Table listing strains and plasmids should be included, listing their source. They mention a JA200 and a recipient MG1655 Sm-resistant strain, where did it come from, which is its genotype? What is plasmid pPGL2134_pgsA_ampR like? They mention in the results that donor strains harbored the "derepressed" RP4 and R27 plasmids, are they using the natural isolates or some mutants? And what about R388 or pKpQIL?

We are including Supplementary Table 2 in the Supplementary Material file that lists all the strains and plasmids used in this study. The pPGL2134_pgsA_ampR is a derivative of pSC101 constitutively expressing *pgsA*. Only R27 is derepressed; it carries an *htdA* mutant (https://doi.org/10.3389/fmolb.2020.00124). R388 and pKpQIL are natural isolates.

b. The conjugation protocol is really odd: 3 hours at 37ºC, then o/n incubation at 25ºC for R27, and 30ºC for RP4. It is particularly surprising since 37ºC is a restrictive temperature for R27, so this plasmid should not be mating under these circumstances. In addition, the donor:recipient ratio is 10 times lower for RP4. What is the rationale for all this?

The 37C incubation is for cell recovery and not for the conjugation experiments. We have amended the Materials and Methods to make it clear.

c. The authors include the results of conjugations with R388 and pKpQIL in Figure 4, but there is no mention to conjugation conditions in MM section (they do not even mention which antibiotic resistance they are using to select them). If RP4 is assayed in different conditions to the other 3 plasmids (with respect to temperature, time of incubation, and especially, donor:recipient ratio), extra controls should be run to discard that the differences in conjugation frequencies are caused by any of these experimental conditions.

We have updated our Materials and Methods to include the conditions for the other plasmids. RP4 was tested under similar conditions as R388 and pKpQIL with the exception of the donor:recipient ratio that is 1:1 for RP4 and R388 but 8:1 for pKpQIL (these ratios are well established for efficient conjugation). See revised Materials and Methods with appropriate references.

d. One reviewer sensibly asked for the CFU data and authors claimed to have included them in the SI, but they are not visible anywhere. This is a relevant information, please do show those data.

We had provided the requested data as a Source Data file in our submission. The data can be found in Source Data file (excel format)

Reviewer #4 (Remarks to the Author):

The revised manuscript by Ishimoto et al. presents additional data supporting the major conclusions. Overall, the manuscript provides novel insights into conjugative pilus biology and function, and presents the first structure of a lipid-independent mating pilus. In response to prior review, the authors have satisfactorily addressed my major concerns with one caveat - based on the presented data, the speculative statement asserting that the absence of phospholipids imparts RP4 pilus rigidity and prevents pilus retraction remains questionable. This point was also raised by Reviewer 2 in the prior review. While the authors provide arguments supporting their assertion, and the new structural information suggests one plausible mechanism that may account for for pilus rigidity and inability to retract, the language in the manuscript should be significantly modified to reflect alternative mechanistic possibilities.

Reviewer #2 raised a similar point. We have tuned down the claims and revised the manuscript accordingly (changes are highlighted in yellow).
